# On the Study of Botany
*We have inserted this lively Essay at the request of a correspondent, who translated it with some alterations from the French of Jondot, in a recent number of the Esprits des Journeaux. Edit.


**Published:** 1811-10

**Authors:** 


### *For the Medical and Physical Journal.*

### *On the Study of Botany.**

SOME individuals will climb the highest mountains, descend into the lowest valleys, and wander on the edge of the most frightful precipices, to discover a few solitary plants to describe in a herbal. There is no disputing about taste; every one is drawn by his pleasure. Botanists will cross seas, traverse desart regions, and brave a thousand dangers, to acquire vegetable productions. Wherever they go, they neither see nor acknowledge any empire but that of Flora. Men, customs, and governments are open to their view, but the leading passion makes them indifferent to the most instructive parts of the moral world. This class of enthusiasts is numerous and powerful; their works are eagerly sought for, and richly patronized.

Natural history, exempt from the absurdities which some of its zealous followers have connected with it, is one of the most agreeable sciences that can be cultivated for amusement. Who can behold without emotion the richness spread with profusion over the country? How beautiful are the commonest flowers! How dazzling their colours! What an astonishing variety they exhibit! Other vegetables, again, have more or less affinity to our wants; wherever we go, even amongst the snows of the Arctic Pole, we find them ornament-

* We have inserted this lively Essay at the request of a correspondent, who translated it with some alterations from the French of Jondot, in a recent number of the Esprits des Journeaux. EDIT.

ing the habitations of man, contributing to his comfort or his luxury.

Let us admire the wisdom of that Providence who, in producing vegetables, has so amazingly diversified their organization. It is the hand of divinity alone that has planted the earth with innumerable buds, whose fruitfulness is inexhaustible; for when one dies, another springs up from its remains; a truth that the younger Racine has well expressed:

> Toute plante, en naissant, renferme en elle

> D'enfans qui la suivront une race immortelle.

Flowers and plants are also worthy our notice, as being the most ancient monuments of our globe; immortal by the laws of reproduction, they survive the destruction of empires, and rise on the wreck of cities. The most successful conqueror cannot destroy them: some Dutch captains attempted once to annihilate several kinds of trees at the Moluccas, to concentrate the harvest of their fruits in the island of Amboyna, but in vain; they exercised their tyranny upon the natives and the plants, their cruelty succeeded with the former, but they could not extirpate the latter; the birds, unconscious of the deed, planted them again. The senseless Europeans declared war against these new enemies and destroyed them; but the winds, which they could not overcome, propagated the seeds of the spices, and they reappeared in spite of the jealousy, the avarice, and the cruelty of the colonists.

The impossibility of discovering the principle of life in plants, is a proof of the weakness of the human mind. In vain have modern ages produced a Tournefort, a Ray, a Cowley, a Vaillant, a Jussieu, a Geoffroi, a Linnæus; these indefatigable observers have found their wisdom baffled by the lichens and the humble hyssop, as much as by the oak or the cedar. The secret of the operations of nature, in the growth, the nourishment, and the unfolding of the least shrub, escapes their knowledge; every thing leads the religious mind, struck with the mysteries of nature, to adore that sublime Being who created so many wonders.

If the vegetable kingdom alone can cause such admiration of the supreme Creator, what will our feelings be when we attempt to consider the whole of his works? The imagination confounded recoils from the infinitude that oppresses it; although we know not the thousandth part of terrestrial productions, those that we are totally unacquainted with are incalculable; the ocean conceals an innumerable multitude in its vast abyss. We regard as fabulous the histories given by the Icelanders and the Norwegians of the kraken and other fishes fishes of huge size, but these fables, however absurd they may appear, are always founded more or less upon truth.

In examining the pursuits of botanists, it is curious to observe the contentions which this science, that of all others ought to inspire a pacific disposition, has caused. For some time they have assumed all the pride and the presumption of men of letters, although the study of flowers and plants simply directed to public utility, contains nothing that is difficult to attain. Every man, with a little patience, is capable of succeeding in it; even though deficient in ability for other sciences, he has before him a career that he may pursue with honour. We have almost from our birth an irresistible propensity to examine the richness and beauty with which the earth is ornamented, and a personal interest which invites us to contemplate it.

Natural history, limited to the study of vegetables, is truly a popular science, and the greatest part of botanists are not essentially different from common gardeners; only the former enjoy the privilege of inventing systems, methods, classes, and composing books: to load each plant with four or five names from the Greek or Latin languages, and to cover them with hieroglyphic characters, which merely serve to embarrass the memory. The gardener calls them by their vulgar names, and is contented with knowing their ordinary properties. The botanists go much farther, they write upon the loves, the manners, the habits, the sleep, the great sensibility of flowers, and discover every day admirable secrets. These gentlemen are as much moved at the sight of a cabbage as at one of their fellow-creatures; and it were to be wished that many of these enthusiasts, deaf to the voice of nature, blind witnesses of the wonders she displays, did not forget the sublime Author of all! Of what use are their extasies at the sight of a plant, since the whole universe is nothing to them but a picture plunged in original darkness.

Linnæus certainly did not give them such an example, for this learned botanist made his reason subservient to the truths of christianity, and was truly religious. There is nothing new under the sun; the fanaticism we have just noticed may be traced back to the remotest antiquity.

Empedocles believed in the sexes of plants, and celebrated with enthusiasm the marriage of flowers; superstition well worthy of a philosopher, who, wishing to be thought a god, threw himself into the burning crater of Ætna.

Without doubt it is necessary to know something of this vast vocabulary of modern botanists, a barrier that must be overcome before we can enter upon vegetable physiology; but mode ought not to be taken for substance, nor the science of of words suppress that of things. Gardeners, manufacturers, and labourers may well be excused from acquiring this learned jargon of Greek and Latin terms. Buffon himself ridicules this wish for giving names which obscure the judgment and render the commonest of all sciences inaccessible. If we desire to know the properties of plants, of what use are those voluminous dictionaries which detail their organic form? Botanists may display the pomp of a ludicrous erudition, but mankind is not benefited by such learned trifling. "The only books that can instruct us," says Fontenelle, "have been spread all over the globe;" let them consult these books, let them clear them of unnecessary things, and penetrate into the only knowledge useful to mankind. Esculapius, Podalirius, Machaon, and Hippocrates of the ancients, did not amuse themselves by inventing systems, nor in composing works on the sex and loves of plants; no, their conduct was more commendable, they consecrated their time to discover the medicinal qualities of vegetables, to find the useful parts, and apply them to the cure of diseases.

In France, as well as in England, the botanists have not to reproach themselves with the ridiculous sentiments and absurd admiration which distinguished those of the last age. They labour unceasingly to increase the natural riches of their country, by introducing a great number of exotic plants. In the one country, the Jardin des Plantes, and in the other, the Garden at Chelsea, will soon inclose an abridgment of the universe, and through the industry of a few individuals, and the protection of the government, we have an opportunity of viewing the productions of all climates.

Contemplating these magnificent treasures of nature, we feel an agreeable surprize, and are most grateful to the authors of these benefits, and to those who are every day endeavouring to collect new objects of curiosity. The ancients never offered a spectacle of this kind to the admiration of the people. They lived always in solitude, and their researches were confined to a country whose natural riches were very limited. When the Romans discovered the cherry-tree, it decorated the triumph of Lucullus, and they were as proud of this discovery as of a victory. If we would form any idea of the poverty of nations with respect to vegetable productions, we must read the Historical and Biographical Sketches of the Progress of Botany in England, translated from the English of Richard Pulteney, by M. B. a worthy member of the republic of letters, and to whom we owe some other good works. We there see how barbarous and neglected was that Gaul, which is now covered with the productions of every country in in the globe. Its extensive forests contained but seven or eight species of trees.

Our situation on the earth is between the infinitely large and the infinitely small, so that we may alternately contemplate the immense concave above our heads and the plants and insects at our feet; whichever of these wonders we examine, God is equally evident and great in all. We may say with Pliny, that natnre is no where more complete than in the smallest beings. (Cum rerum natura nunquam magis, quam in minimis tota sit.) Our proud reason revolts against the miracles of the christian religion, yet even now we tread upon wonders, and though we cannot comprehend them, they still surround us. Who can pierce the mysterious veil with which nature envelopes her operations? See a seed of the linden, though almost imperceptible, it contains an entire tree, whose branches will one day spread over the country, and whose shadow will afford a retreat to an innumerable crowd of living creatures.

The production of plants is one of the most admirable mysteries of divine wisdom. Deprived of the power of moving, they carry on an invisible correspondence. The principles of reproduction float in the atmosphere, from whence arise the sweet perfumes that scent the air. "A bee," writes M. de Chateaubriant," gathers honey from different flowers, and without knowing it makes a whole meadow fruitful; a butterfly carries a whole race in its wings; a world descends in a drop of dew. Animals also are indefatigable agents to the fecundity of plants: the butterfly is by no means an emblem of inconstancy; if it flutters about, it is to carry on a commerce which makes the immense empire of nature flourish." Even children in their play contribute to it involuntarily. They blow on the seeds of the dandelion, which spread sometimes over two or three hundred leagues, and fix themselves on high towers, ramparts, and amidst all the horrors of war.

Such are the pure sentiments of enthusiasm that nature inspires us with. Poets have sung the flowers and metamorphosed them into goddesses. Botany, thus introduced into poetry, furnishes us with the most beautiful mythological allusions, and the most charming images. Rapin, in France, and Cowley, in England, have given scope to their imaginations in the empire of Flora, though reduced to imperfect ideas of natural history: they did not endeavour to make new systems, but to write and work on a subject valuable from its antiquity. But Cowley passed the bounds of reason in celebrating the virtues of plants, and the pretended affinity they had had to man. First he gave them a physiognomy, and then our propensities, our tastes, and our passions.

Since that period, numerous superstitious enthusiasts sought the moral world in the fields, and had no pleasure but in vegetables. We should read the works of certain naturalists to know the dangers and consequences of such exaggeration. In a work that sold very rapidly, we are told, that "Man when asleep is a vegetable." Any simpleton might prove the contrary. The author of this assertion goes much farther, and allows himself the singular pleasure of despising the human race and exalting the brute: "How mean," cries he, "are our societies when compared with nature!" As if man did not form the most beautiful link in the chain of beings spread over the globe! This nature, so much extolled by some philosophers, is an imaginary divinity whom they adore in their writings, with the affectation of overturning the altars of the true divinity.

All the Egyptian superstitions seem to have revived amongst the moderns. Darwin in his poem, translated by M. Deleuze, depicts plants as reasonable beings, discovers qualities in them which would make a sage proud, and introduces us into a round of illusions, not less dangerous than seducing. Some botanists thought they could perceive in their flowers the most amiable and dearest affections of our hearts, and did not hesitate to receive the errors of the Bramins and Pythagoreans. The age of knowledge has revived the idle reveries, with which history so justly reproaches the nations that people the shores of the Indies. In our days we may find a learned man examining the seeds of a plant in the same manner as a mother would look at her children in the cradle. This foolish tenderness shone in society and became fashionable. What they call the (facies externa) or figure of a plant, excited as great transports as the sight of a beautiful woman.

Aristotle, Theophrastes, Dioscorides, and Pliny, amongst the ancients, confined themselves to consider the propagation, the culture and the properties of vegetables, without recurring to those studies where the imagination is the principal guide, nor to those thousands of artificial systems whose falseness is shewn by every increase of experience. And, in fact, what science allows of such direct contradictions in nature, as botany? Some take the fruit to found the classes on, others the flower, others the form of the corolla, and others the calix. Each has his partizans and admirers, and discord rages with as much fury in the empire of Flora as in the political world. Enormous volumes support the arguments of each principle, and all the resources of logic are exhausted to prove that such or such plant plant belongs to the system they approve of. With what warmth do they contend for a simple lichen! and what importance they attach to the least objects!

We must allow, that uncertain as it is, it would be impossible to unite plants by the laws of universal harmony. Botanists fix upon some features which resemble in different productions; but when they flatter themselves that they have filled up the prodigious space between the hyssop and the cedar, they are as much deceived as those naturalists who think they are acquainted with all the intermediate beings between the mite and the elephant. Nature derides their efforts; thousands of links escape them in the chain. The vegetable kingdom is full of obscurity, and they lose themselves in the chaos. We are quite ignorant of the flowers of the north-west shores of America, Chili, indeed the whole of the New World, the interior of Africa, Hindostan, Thibet, Tartary, China, and the Corea, in fact the richest half of Asia; and yet we boast of having the vegetable riches of all the earth, and of seeing the principal works and operations of nature!

It is evident then, that the means actually used are insufficient; they may satisfy our vanity, but not our reason. In proportion as our knowledge increases we must multiply these means so much, that they would exceed the patience of man. The classifications will be deranged, unless the botanists can force nature to bow to their caprice, and treat her as the robber Procrustes treated his visitors. How such a variety and number of new species will increase the catalogues of Linnæus or Jussieu! Perhaps, then, we shall wish that they would again employ themselves in classing plants by climates. Studying them in their native situations would be the best method of composing a kind of geographical botany.

We do not take in the plants that grow in the ocean, the greatest part of which cannot be ranged in any established class. "Sometimes," says Stavorinus, "meadows enamelled with flowers rise to the surface of the sea, and appear covered with verdure." Some of these floating meadows detach themselves from the coast of Norway, and often resting in their way, load themselves with new productions; they stop at America, after having afforded a refuge for thousands of gulls, and a great number of other birds, who, with the assistance of these rafts, perform a voyage of eighteen hundred leagues. The mind of man is incapable of examining all the vegetable productions, as a great part of them are hid at the bottom of precipices, in the midst of desarts, at the top of high mountains, or in the depths of the ocean. Let us not say then that nature has revealed her secrets to us; but let us be contented with enjoying the treasures she has disclosed, and freely confess, that the mysterious veil with which she surrounds herself is impenetrable by our curiosity.

